# Effects of Combined High-Protein Diet and Exercise Intervention on Cardiometabolic Health in Middle-Aged Obese Adults: A Randomized Controlled Trial

**DOI:** 10.3389/fcvm.2021.705282

**Published:** 2021-08-16

**Authors:** Chiao-Nan Chen, Kuo-Jen Hsu, Kuei-Yu Chien, Jeu-Jung Chen

**Affiliations:** ^1^Department of Physical Therapy and Assistive Technology, School of Biomedical Science and Engineering, National Yang Ming Chiao Tung University, Taipei, Taiwan; ^2^Graduate Institute of Sports Science, National Taiwan Sport University, Taoyuan, Taiwan; ^3^Department of Rehabilitation, Taiwan Adventist Hospital, Taipei, Taiwan

**Keywords:** obesity, glucose homeostasis, insulin, oral glucose tolerance test, aerobic exercise

## Abstract

**Background:** Obesity is the main risk factor of cardiovascular diseases (CVD) and metabolic diseases. The middle-aged population is the age group with the highest prevalence of obesity. Thus, improving cardiometabolic health is important to prevent CVD and metabolic diseases in middle-aged obese adults. The aim of this study was to examine the effects of exercise alone or in combination with a high-protein diet on markers of cardiometabolic health in middle-aged adults with obesity.

**Methods:** Sixty-nine middle-aged adults with obesity were assigned randomly to the control group (C; *n* = 23), exercise group (E; *n* = 23), or exercise combined with high-protein diet group (EP; *n* = 23). Individuals in the E and EP groups received supervised exercise training and individuals in the EP group received high-protein diet intervention. Body composition (assessed by dual-energy X-ray absorptiometry), oral glucose tolerance test (OGTT), lipid profiles, and inflammatory markers were determined before and after 12 weeks of intervention. Insulin sensitivity index (ISI_0,120_) was calculated from values of fasting and 2-h insulin and glucose concentration of OGTT. Insulin-peak-time during the OGTT was recorded to reflect β-cell function. Analysis of covariance with baseline values as covariates was used to examine the effects of the intervention. The significant level was set at 0.05.

**Results:** After 12 weeks of intervention, the E group had a greater percentage of individuals with early insulin-peak-time during the OGTT than that in the C and EP groups (*p* = 0.031). EP group had lower total cholesterol and triglycerides than that in the C group (*p* = 0.046 and 0.014, respectively). Within-group comparisons showed that the 2-h glucose of OGTT and C-reactive protein decreased in the EP group (*p* = 0.013 and 0.008, respectively) but not in the E and C groups; insulin sensitivity improved in the EP group (*p* = 0.016) and had a trend to improve in the E group (*p* = 0.052); and abdominal fat mass and total body fat mass decreased in both intervention groups (*p* < 0.05).

**Conclusion:** Combined high-protein diet and exercise intervention significantly decreased fat mass and improved lipid profiles, insulin sensitivity, glucose tolerance, and inflammation in middle-aged adults with obesity.

**Clinical Trial Registration:** Thai Clinical Trials Registry, TCTR20180913003, 13-09-2018.

## Introduction

Obesity is the main risk factor of metabolic diseases, cardiovascular diseases (CVD), and some types of cancer (1–3). The middle-aged population is the age group with the highest prevalence of obesity (4). Therefore, establishing effective evidence-based strategies to decrease CVD risks in the middle-aged population with obesity is a major research priority.

Biomarkers of cardiometabolic health include insulin sensitivity, lipid profiles, and markers of inflammation (5, 6). Increased visceral fat of individuals with obesity causes glucose intolerance, impaired lipid profiles, and greater inflammatory cytokines than individuals with normal weight (7–9). A meta-analysis reported that the risk of CVD mortality increased 13% per 88.5 mg/dl triglycerides (TG) increment (5). Postmenopausal women with the highest levels of total cholesterol (CHOL) combined with the highest levels of C-reactive protein (CRP) were 5 times at risk for CVD than individuals with the lowest levels of CHOL combined with the lowest levels of CRP (10).

Exercise and nutrition are fundamental strategies to improve the cardiometabolic health of individuals. Exercise, especially aerobic exercise, decreases fat mass and inflammation and improves lipid profile and insulin sensitivity in overweight/obese individuals regardless of age (11–13). Calorie restriction is a commonly used diet intervention for individuals with obesity. However, long-term engagement of calorie restriction for most middle-aged individuals is challenging. Recently, increasing evidence demonstrated the benefit of high-protein diet in individuals with obesity. Studies found that high-protein diet (20–30% energy intake from protein) increased satiety and induced sustained reductions in appetite (14, 15). In addition, high-protein diets (≥25% energy intake from protein) have been shown to decrease body weight and fat mass and improve insulin sensitivity and blood pressure in individuals with obesity and patients with diabetes (16, 17).

Collectively, obesity in the middle-aged population requires attention and management to prevent obesity-related diseases. Research has shown that both exercise and high-protein diets have potential to be beneficial for the cardiometabolic health of individuals with obesity (13, 17). While many studies investigating the effects of exercise and nutrition found that high-protein diets/protein supplementation combined with exercise have stronger beneficial effects on skeletal muscle functions and physical functions than exercise alone (18, 19), the knowledge about the effects of exercise alone or in combination with high-protein diet on markers of the cardiometabolic health is lacking. In this regard, this study aimed to examine the effects of exercise alone or in combination with high-protein diet on markers of cardiometabolic health in middle-aged adults with obesity.

## Methods

### Participants

The complete description of participant recruitment and randomization has been previously published (19). Briefly, 69 middle-aged (range: 50–64 years old; mean: 58.3 ± 0.5 years old) individuals (63 females and 6 males) with obesity (body mass index (BMI): 27.7 ± 0.4 kg/m^2^; body fat: 41.5 ± 0.6%; waist circumference: 90.9 ± 1.0 centimeters) were randomly assigned into the control group (C), the exercise group (E) or the exercise combined with high-protein diet group (EP) and received 12 weeks of intervention. The prevalence of hypertension and type 2 diabetes mellitus (T2DM) was similar among the 3 groups (~1/3 hypertension, and 1/5 T2DM). Participants in the EP group had a 4 times higher prevalence of dyslipidemia (35%) than participants in the C (9%) and E groups (9%). This study was approved by the Institutional Review Board of the National Yang-Ming University and all the participants signed the consent form (IRB number: YM-106064F-1).

### Exercise Intervention

**Figure 1:**
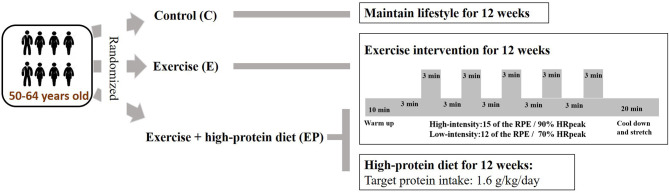
Study design.

As shown in the Figure 1, individuals in the E and EP groups received supervised exercise training on the spin bikes in a fitness room. The duration of each session was 60 min, including 10 min of warm-up, 30 min of high-intensity interval training (HIIT), and 20 min of cool-down and stretching. High-intensity interval training consisted of 5 cycles of 3-min of high-intensity exercise and 3-min of low-intensity exercise. The high-intensity was set at 90% of HR_peak_ or at 15 of the Borg 6-to-20 rating of perceived exertion (RPE) scale. The low-intensity was set at 70% HR_peak_ or at 12 of the RPE scale. A graded exercise test on a cycle ergometer with a metabolic system (Cortex Metalyzer 3B, Germany) was used to determine the HR_peak_. Heart rate monitors (Polar RS400, USA) were used to ensure participants exercised at the target exercise intensity. The average attendance rate of exercise training of the E and EP groups was 90 and 94%, respectively. The average HR at the high-intensity component of HIIT was 91% HR_peak_ and 90% HR_peak_ for the E and the EP groups, respectively. The attendance rates and training intensity were similar between the E and the EP groups.

### Diet Intervention

**Figure 2:**
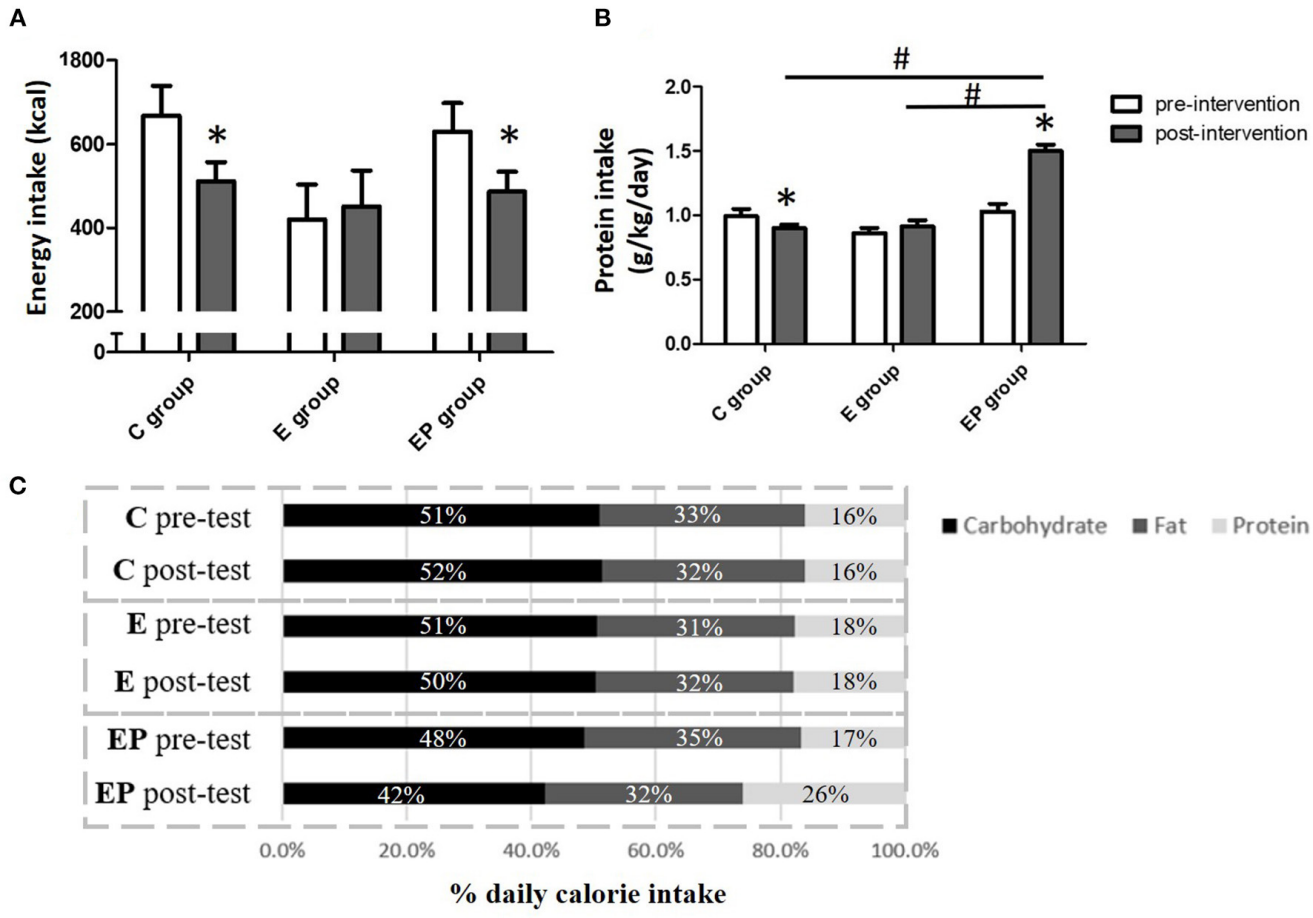
Daily energy intake of participants before and after intervention. **(A)** Daily energy intake. **(B)** Daily protein intake. **(C)** The percentage of macronutrients of daily energy intake. Data were presented as mean ± SE or percentage. C, control group; E, exercise group; EP, exercise combined with high-protein diet group. *Significant change from the baseline. ^#^Significantly different between groups.

The target protein intake of this study was 1.6 g/kg/day, which is higher than the current recommended dietary allowance (RDA) of protein for adults at 0.8 g/kg/day (20). Daily protein intake at 1.6 g/kg body weight (BW) was chosen because 1.6 g/kg/day of protein intake was shown to protect lean mass in response to exercise– and/or dietary-induced weight loss and to maximize the increase of muscle mass in healthy adults with exercise (21, 22). In the current study, whey protein powder supplements (M Power All WHEY plain, BriPower International Enterprise, Taiwan) was provided to participants to help them reach the target daily protein intake because our pilot study found that it was difficult for Asian middle-aged people to achieve 1.6 g/kg/day of protein intake with the traditional diet. The whey protein supplementation contained 50% of essential amino acid and 50% of non-essential amino acid. It provided 20 g of branch-chain amino acid (46% of leucine, 28% of isoleucine, and 26% of valine) per 100 g of protein. The target calorie intake was 25 kcal/kg body weight (Health Promotion Administration, Ministry of Health, and Welfare, Taiwan). Participants in the EP group were asked to record their daily food and beverage intake and upload the information to an application (Cofit, Cofit Healthcare Inc., Taiwan). A licensed dietitian analyzed the nutritional composition of the dietary intake of participants based on the report that participants uploaded and provided the diet education and consultation for participants. The dietitian also calculated the insufficient protein intake and gave the appropriate amount of protein powder so that participants could reach the target protein intake of the study. At baseline, the average daily energy and protein intakes were not significantly different among groups. Diet intervention increased the protein intake of participants in the EP group to the level of 1.5 ± 0.05 g/kg/day. In addition, individuals in the EP group had greater percentage of energy intake from protein and lower percentage of energy intake from carbohydrate than that in the C and the E groups (Figure 2).

### Outcome Measurement

#### Body Composition

Body height and body weight were assessed before and after the 12-week intervention. Body composition (fat mass and muscle mass) was assessed by dual-energy X-ray absorptiometry (DXA) (Lunar iDXA, GE Medical System, Madison, WI).

#### Oral Glucose Tolerance Test

Oral glucose tolerance test (OGTT) challenges glucose homeostasis and reflects insulin sensitivity and β-cell function of individuals (23, 24). Oral glucose tolerance test was performed on participants without diabetes mellitus (DM) according to the recommendation of the World Health Organization. Specifically, individuals drank 300 ml of water with 75 g of glucose in 5 min after overnight fasting. Blood samples were taken at baseline and at 30, 60, and 120 min after drinking the glucose water. Plasma glucose and serum insulin levels were determined by the hexokinase glucose-6-phosphate dehydrogenase (G-6-PDH) method and the immunoradiometric assay, respectively. The area under curves (AUCs) of glucose and insulin levels at different time points were calculated by the trapezoid method.

The insulin-peak-time was determined as the time point that insulin concentration reached its highest value during the OGTT. The insulin-peak-time reflects the function of beta cells (25, 26). In addition, insulin sensitivity index_0,120_ (ISI_0,120_) was calculated by the following formula (27):

$$\begin{array}{l} \text{ m}=[75,000\text{ mg}+(\text{fasting glucose}\\ \;-120\;\min\text{ glucose})\times 0.19\\ \;\times \text{BW}]÷120\\ \text{ Mean plasma glucose}(\text{MPG})=(\text{fasting glucose}\\ \;+120\;\min\text{ glucose})÷2\\ \text{ Mean serum insulin}(\text{MSI})=\log[(\text{fasting insulin}\\ \;+120\;\min\text{ insulin})÷2]\\ \text{Metabolic clearance rate}(\text{MCR})=\text{m}÷\text{MPG}\\ {\text{ ISI}}_{0,120}=\text{MCR }÷\text{MSI} \end{array}$$

Insulin sensitivity index_0,120_ has been shown to be a valid index of insulin sensitivity in various populations and has been used in many clinical studies (27–29). The higher values of the index indicate the better insulin sensitivity of individuals.

#### Lipid Profiles

Participants arrived laboratory at 8:00 a.m. after a 12-h overnight fast and 2 ml of venous blood was collected. Fasting serum CHOL, TG, low-density lipoprotein (LDL), and high-density lipoprotein (HDL) were analyzed at baseline and after the 12-weeks of intervention. All lipid content measurements were performed by the Beckman Coulter AU5820 (Beckman Coulter, Inc., Brea, CA, USA) with 300 μl of serum. Each test was performed according to the manufacturer's protocol (30, 31). The intra-assay coefficients of variation for TG, CHOL, LDL, and HDL were <2.9, <2.6, <4.7, and <5.0%, respectively. The CHOL to HDL ratio and the LDL to HDL ratio were calculated. Greater risk of cardiovascular diseases/events have been shown to be associated with higher ratio values of CHOL to HDL and LDL to HDL (32, 33).

#### Markers of Inflammation and Oxidative Stress

Plasma levels of C-reactive proteins were determined by the ELISA-kit (#378020 from Beckman Coulter, Brea, Calif., USA). Briefly, plasma samples (50 μl) were incubated with CRP Latex Reagent and analyzed on AU5820 auto-analyzer (Beckman Coulter, Inc., Brea, CA, USA). The intra-assay coefficients of variation (CV) for CRP was <3.0% (34).

Oxidative stress was determined by the superoxide dismutase activity in red blood cells (SOD-RBC). The RBC were lysed with four-times volumes of ice-cold HPLC-grade deionized water and centrifuge at 10000 g for 15 min at 4°C. The erythrocytes lysate was incubated in an assay medium containing phosphate buffer and the reaction was initiated by adding xanthine oxidase. The activity of SOD was determined spectrophotometrically at a wavelength of 450 nm for 20 min at 37°C. The optical density (OD) was determined by a spectrophotometer (Tecan, Männedorf, Switzerland) (35).

### Statistical Analysis

Data were presented as mean ± standard error (SE). All outcome variables were tested for normal distribution by the Shapiro-Wilk test. Fasting insulin concentration, TG, LDL to HDL ratio, CHOL to HDL ratio, and CRP were log-transformed before the statistical analysis because data were not normally distributed. Differences in baseline characteristics among groups were determined by one-way analysis of variance (ANOVA) for continuous variables and by the chi-square test for categorical variables. Treatment effects were determined by analysis of covariance (ANCOVA) with baseline values as covariates. The Bonferroni test was used as the *post-hoc* test. Within-group comparisons were performed with paired *t*-test. An intention to treat analysis was used in this study where missing data were inputted with pre-test values based on the last-observation-carried-forward technique. Pearson's correlation coefficients were used to determine the relationship between daily protein intake and outcome variables. The G*power was used to calculate the needed sample size and 22 participants were required for each group with a statistical power of 0.95, effects size of 0.25, and alpha value of 0.05. The significance level was set at *p* < 0.05. SPSS 24.0 (Statistical Package for the Social Sciences, SPSS, Chicago, USA) was used to perform statistical analyses.

## Results

### Basic Characteristics of Participants

Overall, the average fasting glucose, fasting insulin, and ISI_0,120_ of all participants was 94.2 ± 1.3 mg/dl, 13.4 ± 0.9 μIU/ml, and 19.9 ± 1.2, respectively. The average CHOL, TG, HDL, and LDL of all participants was 201.8 ± 4.2, 111.8 ± 7.8, 55.2 ± 1.4, and 123.0 ± 4.1 mg/dl, respectively. The average CRP and SOD-RBC of all participants was 0.22 ± 0.02 mg/dl and 94.1 ± 1.3 U/mg-protein. Baseline characteristics related to body composition, glucose homeostasis, lipid profiles, systematic inflammation, and oxidative stress were similar among groups, except for the 2-h glucose of OGTT. Two-hour glucose of OGTT in the E group was lower than that in the EP group (*p* < 0.05).

### Effects of Interventions on Body Composition

Obesity is the main risk factor of metabolic and cardiovascular diseases (2). All parameters including BW, BMI, abdominal fat mass (AFM), total body fat mass (TBF), appendicular skeletal muscle mass (ASM), and total skeletal muscle mass (TSM) were similar among groups after intervention (*p* > 0.05). Within-group comparisons showed that all parameters of body composition did not change in the C group from the pre-test to the post-test. After the intervention, the average BW decreased 1.6% in the EP group; the average BMI decreased from 1.6% in the EP group; AFM decreased 3.6 and 3.7% in the E and EP groups, respectively (*p* < 0.05); TBF decreased 2.8 and 3.0% in the E and EP groups, respectively (*p* < 0.05). The changes of skeletal muscle mass before and after the intervention were not significant in all groups (Table 1 and Figure 3).

**Table 1:** Effects of interventions on anthropometry and body composition.

	**C (** ***n*** **= 23; Male = 2, Female = 21)**		**E (** ***n*** **= 23; Male = 3, Female = 20)**		**EP (** ***n*** **= 23; Male = 1, Female = 22)**	
	**Pre**	**Post**	**Pre**	**Post**	**Pre**	**Post**
**Anthropometry**						
Body height (cm)	157.4 ± 1.3		159.6 ± 1.9		156.9 ± 1.3	
Male	168.8 ± 5.8		173.5 ± 0.5		170 ± 0.0	
Female	156.4 ± 1.1		157.5 ± 1.7		156.1 ± 1.3	
BW (kg)	68.7 ± 2.2	68.4 ± 2.2	71.8 ± 2.5	71.0 ± 2.5	67.1 ± 2.3	66.0 ± 2.1^*^
Male	85.9 ± 11.6	83 ± 7.8	91.8 ± 4.7	91.4 ± 5.0	71.3 ± 0.0	68.5 ± 0.0
Female	67.1 ± 1.9	67.0 ± 2.1	68.8 ± 2.1	68.0 ± 2.0	66.9 ± 2.4	65.9 ± 2.2
BMI (kg/m^2^)	27.6 ± 0.7	27.5 ± 0.7	28.1 ± 0.7	27.8 ± 0.7	27.3 ± 0.9	26.8 ± 0.8^*^
Male	30.0 ± 2.0	29.1 ± 0.8	30.5 ± 1.6	30.4 ± 1.7	24.7 ± 0.0	23.7 ± 0.0
Female	27.4 ± 0.7	27.4 ± 0.8	27.7 ± 0.7	27.4 ± 0.7	27.4 ± 0.9	27.0 ± 0.8
Percentage body fat (%)	41.7 ± 1.0	40.9 ± 1.0	42.7 ± 1.2	42.0 ± 1.1	40.3 ± 0.9	39.9 ± 1.1
Male	32.6 ± 0.3	30.7 ± 1.3	33.3 ± 2.1	33.2 ± 2.0	31.2 ± 0.0	27.6 ± 0.0
Female	42.6 ± 0.9	41.9 ± 0.8^*^	44.1 ± 1.0	43.3 ± 1.0^*^	40.8 ± 0.9	40.5 ± 1.0
**Body composition**						
AFM (kg)	2.4 ± 0.1	2.4 ± 0.1	2.6 ± 0.1	2.5 ± 0.1^*^	2.3 ± 0.1	2.2 ± 0.1^*^
Male	2.8 ± 0.4	2.4 ± 0.0	3.1 ± 0.4	3.0 ± 0.4	2.3 ± 0.0	1.8 ± 0.0
Female	2.4 ± 0.1	2.4 ± 0.1	2.6 ± 0.2	2.5 ± 0.1^#^	2.4 ± 0.2	2.3 ± 0.1^*^
TBF (kg)	27.7 ± 1.1	27.2 ± 1.1	29.8 ± 1.3	28.9 ± 1.2^*^	26.6 ± 1.4	25.8 ± 1.4^*^
Male	27.0 ± 3.6	24.4 ± 0.1	29.7 ± 2.8	29.4 ± 2.9	21.4 ± 0.0	18.2 ± 0.0
Female	27.8 ± 1.1	27.5 ± 1.2	29.8 ± 1.4	28.8 ± 1.4^*^	26.8 ± 1.4	26.1 ± 1.4^*^
ASM (kg)	16.8 ± 0.8	17.0 ± 0.7	17.4 ± 1.0	17.4 ± 1.0	16.7 ± 0.6	16.6 ± 0.6
Male	26.0 ± 4.0	25.5 ± 3.1	27.8 ± 0.8	27.5 ± 1.1	22.4 ± 0.0	22.5 ± 0.0
Female	15.9 ± 0.5	16.2 ± 0.5	15.9 ± 0.5	15.9 ± 0.5	16.4 ± 0.6	16.3 ± 0.6
TSM (kg)	38.9 ± 1.6	39.2 ± 1.5	40.0 ± 1.8	40.1 ± 1.8	38.8 ± 1.3	38.3 ± 1.2
Male	55.9 ± 8.2	55.6 ± 6.5	59.1 ± 2.9	59.0 ± 3.0	47.1 ± 0.0	47.6 ± 0.0
Female	37.3 ± 1.1	37.6 ± 1.1	37.2 ± 0.9	37.3 ± 0.9	38.4 ± 1.2	37.9 ± 1.1

*Data were presented as mean ± SE. AFM, abdominal fat mass; ASM, appendicular skeletal muscle mass; BMI, body mass index; C, control group; E, exercise group; EP, exercise combined with high-protein diet group; TBF, total body fat mass; TSM, total skeletal muscle mass*.

* *Significant change from the baseline*.

# *A trend to change significantly from the baseline (0.1 > p ≥ 0.05)*.

**Figure 3:**
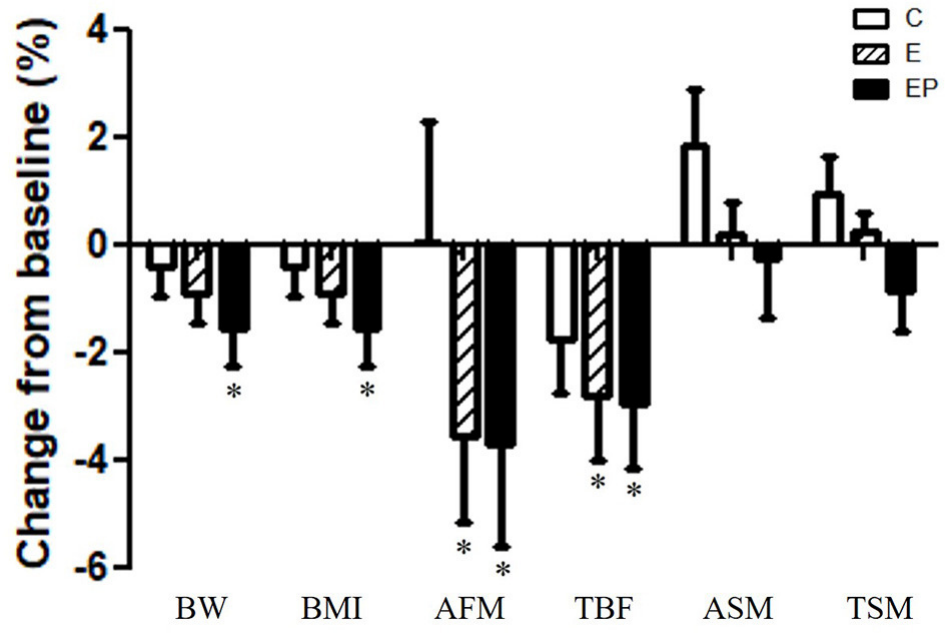
Effects of intervention on body composition. Data were presented as mean ± SE. *Significant change from the baseline; AFM, abdominal fat mass; ASM, appendicular skeletal muscle mass; BMI, body mass index; BW, body weight; C, control group; E, exercise group; EP, exercise combined with high-protein diet group; TBF, total body fat mass; TSM, total skeletal muscle mass.

### Effects of Interventions on Glucose Homeostasis

Glucose homeostasis is a key marker of cardiometabolic health of individuals (36). We found that fasting glucose levels, fasting insulin levels, and AUCs of glucose and insulin were similar among groups after intervention (*p* > 0.05). Within-group comparisons showed that the 2-h glucose of OGTT decreased 10% in the EP group after intervention (*p* < 0.05), but the values did not change in the C and E groups after intervention (*p* > 0.1). The ISI_0,120_ increased 11.5% in the EP group and had a trend to increase in the E group (*p* = 0.052) (Table 2). Regarding the insulin-peak-time, the percentages of individuals having insulin-peak-time at 30, 60, or 120 min of OGTT were different among groups after intervention (*p* = 0.031). In the E group, the percentage of individuals having insulin-peak-time at 30 min increased from 25 to 50%, and the percentage of individuals having insulin-peak-time at 120 min decreased from 37.5 to 12.5% after the intervention. The pattern of insulin-peak-time did not change in the C and EP groups (Figure 4).

**Table 2:** Effects of interventions on variables derived from oral glucose tolerance test.

	**C**		**E**		**EP**	
	**Pre**	**Post**	**Pre**	**Post**	**Pre**	**Post**
Fasting glucose (mg/dl)	94.5 ± 1.7	93.8 ± 1.8	92.8 ± 1.9	92.9 ± 1.8	95.5 ± 3.2	95.2 ± 2.5
Fasting insulin (μIU/ml)	13.1 ± 1.6	11.0 ± 0.7	15.0 ± 1.8	14.9 ± 1.2	11.8 ± 1.0	12.5 ± 1.3
2-h glucose (mg/dl)	186.5 ± 21.6	168.7 ± 17.2	133.8 ± 10.4	128.8 ± 10.7	182.9 ± 14.0	164.1 ± 15.6^*^
AUCs of glucose	19787.5 ± 1561.0	20332.5 ± 1736.6	16872.7 ± 925.1	16412.3 ± 881.5	20940.0 ± 1357.2	20579.3 ± 1176.0
AUCs of insulin	12761.0 ± 792.0	11557.5 ± 996.6	13340.4 ± 1754.2	12041.8 ± 1895.4	9433.8 ± 796.5	10033.5 ± 838.4
ISI_0,120_	17.4 ± 3.5	19.0 ± 2.6	23.3 ± 1.9	25.7 ± 2.3^#^	18.3 ± 1.6	20.4 ± 1.9^*^

*Data were presented as mean ± SE. 2-h, 2 hours. AUCs, area under curves; C, control group; E, exercise group; EP, exercise combined with high-protein diet group; ISI, insulin sensitivity index*.

* *Significant change from the baseline*.

# *A trend to change significantly from the baseline (0.1 > p ≥ 0.05)*.

**Figure 4:**
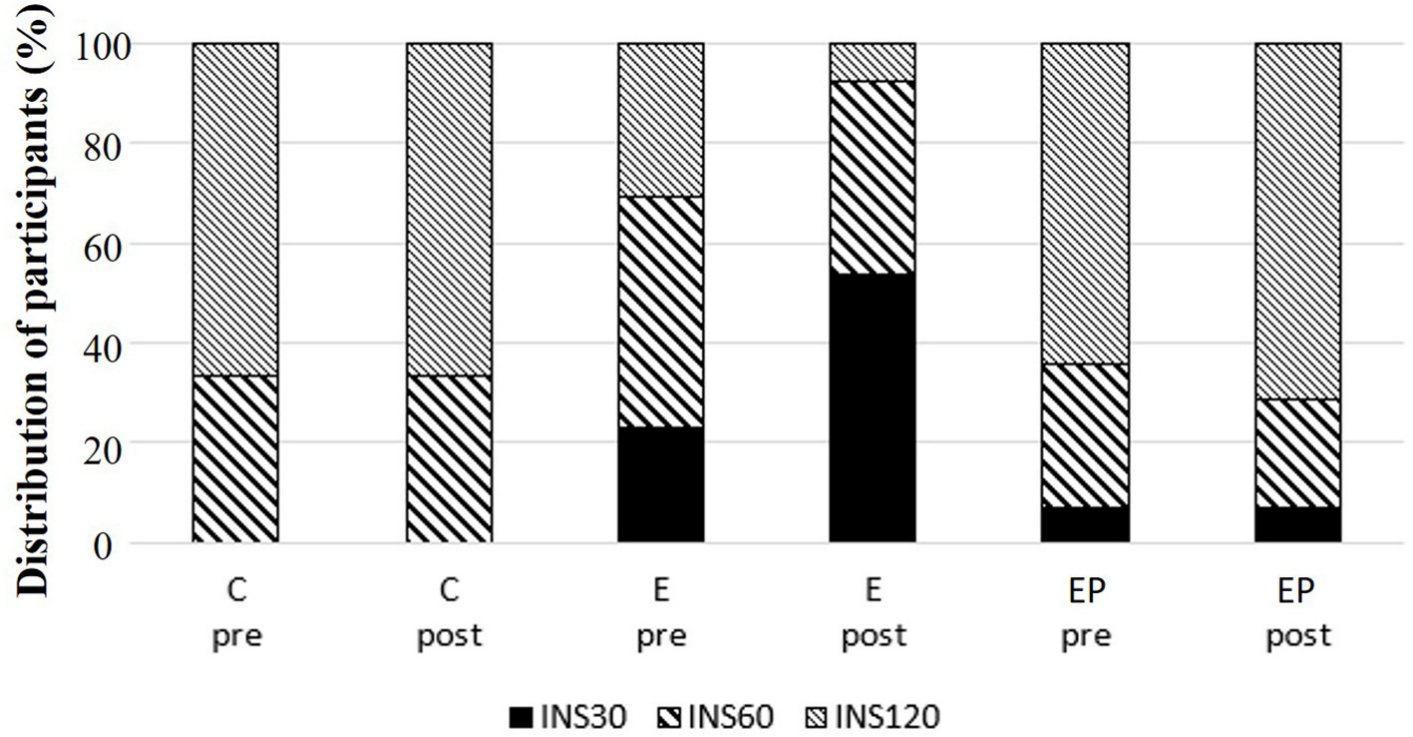
Distribution of insulin-peak-time during oral glucose tolerance test (OGTT) among groups. C, control group; E, exercise group; EP, exercise combined with high-protein diet group. INS30: peak insulin level at 30 min of OGTT. INS60: peak insulin level at 60 min of OGTT. INS120: peak insulin level at 120 min of OGTT.

### Effects of Interventions on Lipid Profiles, Systemic Inflammation, and Oxidative Stress

**Figure 5:**
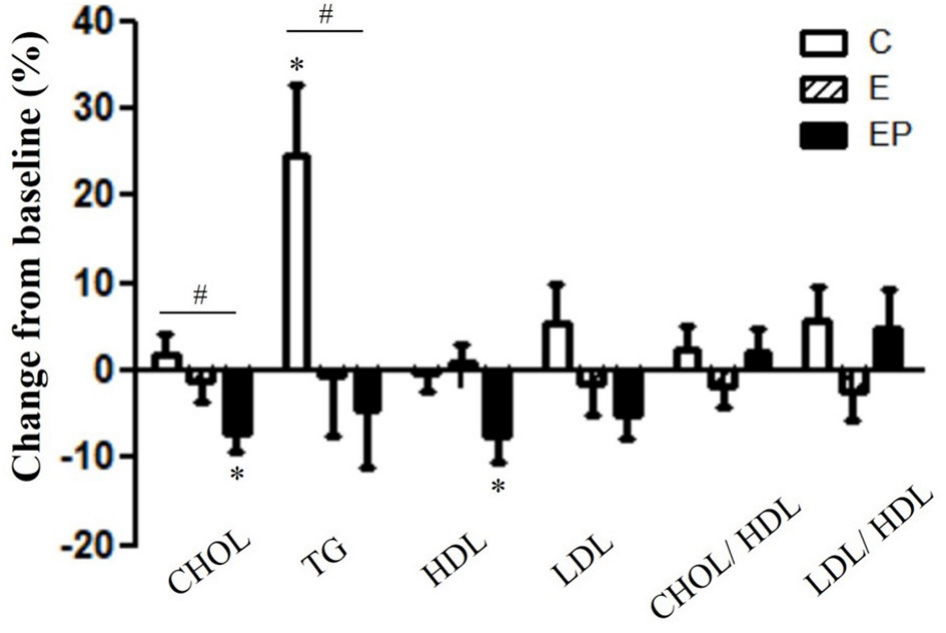
Effects of intervention on lipid profiles. Data were presented as mean ± SE. ^#^Significantly different between groups analyzed by ANCOVA and the Bonferroni test; *Significant change from the baseline. C, control group; CHOL, total cholesterol; E, exercise group; EP, exercise combined with high-protein diet group; HDL, high-density lipoprotein; LDL, low-density lipoprotein.

Dyslipidemia predicts the CVD risk (5, 10). After 12 weeks of intervention, the CHOL and TG of the EP group were significantly lower than that of the C group (*p* < 0.05). The CHOL in the EP group decreased from 204.1 ± 9.4 to 190.2 ± 10.7 mg/dl and it did not change in the C and E groups. The TG value in the C group increased from 101.2 ± 10.9 to 119.0 ± 10.3 mg/dl; whereas, it decreased from 132.4 ± 17.9 to 125.9 ± 17.6 mg/dl in the EP group. There were no group differences in HDL, LDL, the LDL to HDL ratio, and the CHOL to HDL ratio. Within-group comparisons showed that the HDL level decreased after intervention in the EP group (from 55.8 ± 2.8 to 51.1 ± 2.5 mg/dl) (Figure 5).

**Figure 6:**
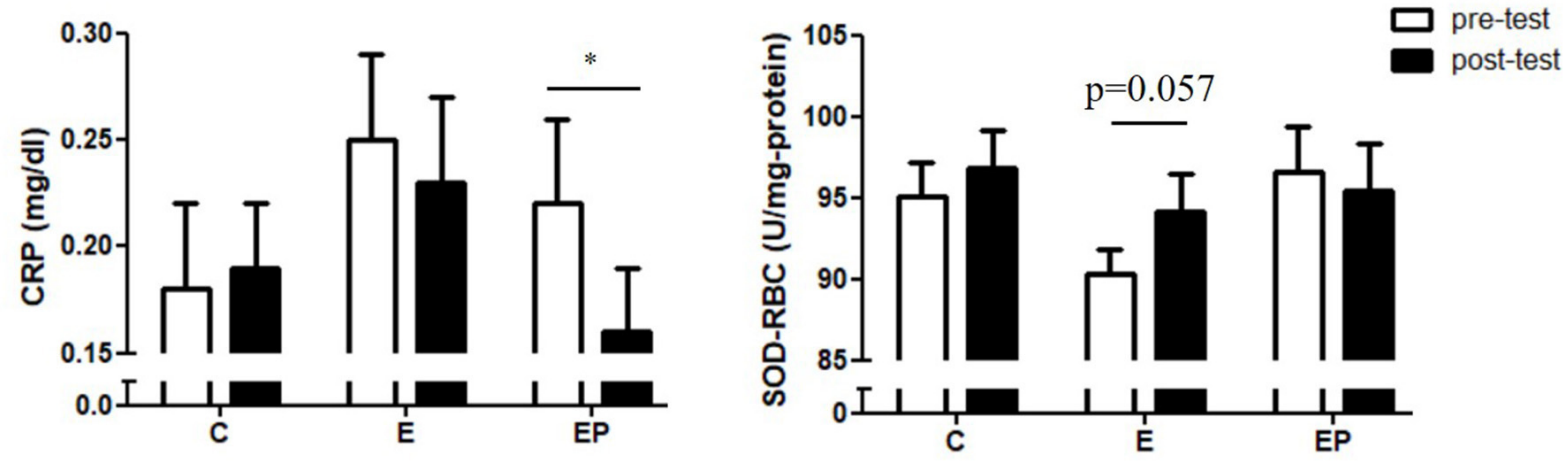
Effects of intervention on inflammatory and oxidative stress markers. Data were presented as mean ± SE. C, control group; CRP, C-reactive protein; E, exercise group; EP, exercise combined with high-protein diet group; SOD-RBC, superoxide dismutase activity in red blood cells. *p < 0.05.

Inflammation and oxidative stress are associated with insulin resistance and vascular dysfunction (37). We found that CRP in the EP group decreased significantly (*p* = 0.008) from 0.22 ± 0.04 to 0.16 ± 0.03 mg/dl. The CRP values in the C and E group did not change significantly. The pre-test and post-test values of the CRP in the C group were 0.18 ± 0.04 and 0.19 ± 0.03, respectively. The pre-test and post-test values of the CRP in the E group were 0.25 ± 0.04 and 0.23 ± 0.04, respectively. In terms of antioxidant SOD-RBC, the level of SOD-RBC in the E group showed a tendency to increase (*p* = 0.057) after intervention. The levels of SOD-RBC in the C and EP group did not change significantly (Figure 6).

### Correlation Between Daily Protein Intake and Outcome Measurements

We found that daily protein intake during the intervention was negatively correlated with BW (*r* = −0.294; *p* = 0.015), BMI (*r* = −0.252; *p* = 0.038), TBF (*r* = −0.351; *p* = 0.003) and AFM (*r* = −0.362; *p* = 0.002) at post-test. There were no significant correlations between daily protein intake and parameters of glucose homeostasis, lipid profiles, and CRP.

## Discussion

This study examined the effects of exercise alone or in combination with high-protein diet on biomarkers of cardiometabolic health in middle-aged obese adults. The main finding is that exercise combined with high-protein diet significantly improved insulin sensitivity (IS) and decreased 2-h glucose of OGTT, CHOL, TG, and CRP levels in middle-aged obese adults. Exercise alone improved β-cell function characterized by the insulin-peak-time during the OGTT. Last, exercise reduced the fat mass of middle-aged obese adults with or without high-protein diet.

Appropriate insulin secretion and IS maintain the glucose homeostasis of individuals. In the current study, we found that IS improved significantly in the EP group and had a trend to improve in the E group. In addition, glucose levels at 2 h of the OGTT decreased 10.3% in the EP group after the intervention but the change in the E group was not significant. Both exercise and high-protein diet have been shown to improve the IS of individuals with obesity (17, 28, 38). However, mechanisms underlying the beneficial effects of exercise and high-protein diet on IS of obese individuals are different. Exercise increases IS by muscle contraction-induced increase of cellular glucose transporters (39). In contrast, high-protein diets increase IS *via* loss of body weight and insulinotropic effects of dietary protein (14, 40). Consistent with previous reports, we found daily energy intake decreased in our participants within the EP group. The BW and BMI also decreased significantly in the EP group. Our finding, the negative correlation between the daily protein intake and BW, BMI, TBF, and AFM, further supports the role of high-protein diet on loss of body weight. The high-protein diet associated greater satiety and reductions in appetite are likely the underlying mechanisms contributing to the reduced energy intake of participants in the EP group (14, 15). Thus, our findings together with others suggest that individuals can take advantage of the specificity of exercise and high-protein diet on the IS pathway to achieve the best outcomes of IS and metabolic health.

To our knowledge, this is the first study examining the effects of exercise and diet on the insulin-peak-time during the OGTT at the same time. Insulin-peak-time during the OGTT reflects β-cell function of individuals (9, 26, 41, 42). Impairment of early-phase insulin release suggests defects of insulin secretion in response to glucose. The delayed and blunted early-phase insulin response to glucose results in reduced suppression of hepatic glucose production and insufficient muscle glucose uptake (43). Praveen et al. (44) reported that individuals (with normal glucose tolerance) whose insulin–peak–time was at 60 min and at 120 min during OGTT had higher postprandial glucose levels than individuals whose insulin-peak-time was at 30 min. Sun et al. (25) followed individuals (with normal glucose tolerance at baseline) over a 6 years period and found that individuals with delayed insulin-peak-time during OGTT at baseline (120 and 180 min) were at a greater risk (7.3 times) for diabetes incidence compared to individuals with shorter insulin-peak-time (30 min) at baseline. In the current study where women were the majority (91%), we found that the number of participants having insulin peak at 30 min during the OGTT doubled in the E group after intervention. The beneficial effects of exercise on β-cell function were also reported in healthy men and men with pre-diabetes or T2DM (45). Although, clear mechanistic details of this phenomenon are still not understood, exercise-associated decreases of pancreatic fat are suggested as a mechanism underlying the exercise-induced benefits on β-cell function (45).

We did not see changes in the pattern of insulin-peak-time during OGTT in the EP group. This finding is unexpected. The non-significant change is likely due to a trend of poorer baseline glucose tolerance in participants of the EP group than that in the E group (*p* = 0.057). Supporting this explanation, Heiskanen et al. (45) demonstrated that exercise training increased early-phase insulin secretion rate in healthy men but not in pre-diabetic/T2DM men. Another possible explanation for the non-significant change of the insulin-peak-time during OGTT in the EP group is the protein source. Kahleova et al. (46) found that with matched energy and macronutrient composition, a meal with plant-based protein induced greater rate sensitivity (insulin secretion rate in response to the change of glucose) than a meal with meat-based protein in men with T2DM. Since the source of protein was not investigated and controlled in this study, the non-beneficial effects of exercise on insulin-peak-time during the OGTT in the EP group could be due to the increased intake of meat. Further, studies are needed to test the hypothesis.

We found that lipid profile did not significantly change in the E group, but CHOL and TG significantly reduced in the EP group. Triglyceride values have been shown to have a moderate and high association with coronary heart diseases (47). A meta-analysis of 160 randomized controlled trials (7,487 participants) showed that while exercise improved lipid profiles, the effects were gender-specific and dependent on the health condition of individuals (38). Greater exercise-related improvement in lipid profiles was reported in men than in women and in individuals with at least one health condition (T2DM, hypertension, hyperlipidemia, and metabolic syndrome) than individuals with no health condition stated above. Thus, there are two possible contributing factors to the non-significant change in lipid profile of the E group: (1) the composition of the study participants was 91% women, and (2) 61% of participants did not present with any of the identified health conditions stated above.

The decrease in TG level with high-protein diet is observed across many studies, including the current study. For instance, a meta-analysis of randomized controlled trials found that 12 weeks of high-protein diets (average protein intake: 1.3 g/kg/day; the average percentage calorie intake from protein: 30%) provided more favorable changes in TG than iso-calorically standard-protein diets (average protein intake: 0.7 g/kg/day; the average percentage calorie intake from protein: 18%) in overweight/obese adults (48). The mechanism underlying the decrease of TG with high-protein diets is likely due to the reduction of the intake of carbohydrates (CHO). In fact, it was shown that when the CHO intake accounts for more than 50% of total calorie intake, there is an elevation in blood TG level (49). Supporting the link between CHO intake and TG level, we demonstrated that the percentage of calorie intake from CHO in the EP group (42%) was significantly lower than that in the C (52%) and E group (50%). Similarly, in the meta-analysis study by Wycherley, the percentages of calorie intake from CHO in the high-protein-diets group and the iso-calorically standard-protein-diets group were 42 and 57% respectively (48). Thus, our findings together with others suggest that high-protein diet associated reduced CHO intake contributes to the reduction of TG levels.

Interestingly, we found HDL did not change in the E group and it decreased in the EP group after intervention. Most studies demonstrated an increase in HDL after exercise training (38, 50–52). A meta-analysis showed that aerobic exercise training with an average duration of 22 weeks significantly increased HDL level in women (50). Thus, the relatively short intervention period (12 weeks) might be a reason why we did not find the increase of HDL after exercise training. Another possible reason for the non-significant effects of exercise on HDL is that participants in this study had normal HDL levels at baseline (55.2 mg/dl). Most studies that found significant increase of HDL with exercise training had subjects with metabolic syndrome or low baseline HDL levels (53, 54). Similar to our finding, Keating et al. (55), showed that exercise training did not increase HDL in adults with normal HDL levels. It is unclear why HDL levels decreased significantly in the EP group, but it could be associated with the changes of HDL subpopulation profile. It was shown that large HDL particles are related with lower CVD risk, whereas, medium and small HDL particles are related with greater CVD risk (56, 57). Thus, further research that analyzes the HDL subpopulation profile is needed to clarify the effects of combined high-protein diet and exercise intervention on middle-aged obese adults. Importantly, although, we found HDL decreased in the EP group after intervention, the CHOL to HDL ratio and the LDL to HDL ratio (two better predictors for CVD morbidity than HDL alone) (32, 58, 59) did not change. This finding suggests that combined high-protein diet and exercise intervention does not increase the CVD risk.

We found that only exercise combined with high-protein diet decreased CRP of middle-aged obese adults; exercise alone did not significantly decrease CRP in this population. To date, the effects of exercise on inflammation are still inconclusive. A meta-analysis showed beneficial effects of exercise on the inflammatory status (CRP and interleukin-6) in adults with obesity (60). In contrast, another meta-analysis showed that exercise (both moderate- and vigorous-intensity) did not improve inflammatory status (CRP, tumor necrosis factor-alpha and interleukin-6) of sedentary healthy adults (38). Previous studies indicated that the decrease of systemic inflammation is associated with the degree of weight loss such that the more weight loss, the more decrease of systemic inflammation (61–63). Thus, the significant decrease of CRP in the EP group but not in the E group is likely associated with greater loss of BW in the EP group. Regarding the important role of the systemic inflammation in the cardiometabolic health, our finding implies that middle-aged obese adults could be suggested to have a high-protein diet in addition to exercise to help loss weight and improve the cardiometabolic health.

Some limitations exist in this study. First, we did not record the source of protein (plant or animal), thus, findings of this study can only suggest levels of daily protein intake. Second, only one oxidative stress marker was evaluated. A more comprehensive assessment including levels of multiple antioxidant enzymes (RBC-SOD, catalase, and glutathione peroxidase), free radical levels, and oxidative damage markers (such as serum malondialdehyde) is needed to provide solid evidence about the effects of intervention on oxidative stress of middle-aged obese adults. In summary, exercise combined with high-protein diet, but not exercise alone, improved IS, glucose tolerance, lipid profiles, and inflammation status in middle-aged obese adults. Thus, high-protein diet can be suggested to middle-aged obese adults who exercise to enhance cardiometabolic health.

## Data Availability Statement

The raw data supporting the conclusions of this article will be made available by the authors, without undue reservation.

## Ethics Statement

The studies involving human participants were reviewed and approved by Institutional Review Board of the National Yang-Ming University. The patients/participants provided their written informed consent to participate in this study.

## Author Contributions

C-NC and K-YC conceptualized the study, analyzed and interpreted the data, and wrote the manuscript. C-NC and K-JH coordinated and managed the project. K-JH and J-JC performed the intervention and collected the data. All authors reviewed and approved the manuscript.

## Conflict of Interest

The authors declare that the research was conducted in the absence of any commercial or financial relationships that could be construed as a potential conflict of interest.

## Publisher's Note

All claims expressed in this article are solely those of the authors and do not necessarily represent those of their affiliated organizations, or those of the publisher, the editors and the reviewers. Any product that may be evaluated in this article, or claim that may be made by its manufacturer, is not guaranteed or endorsed by the publisher.
